# A retrospective single-center cohort study of major subtypes of primary glomerular diseases (MN, IgAN, and MCD): clinical characteristics, prognostic outcomes, and risk factors

**DOI:** 10.3389/fmed.2025.1741853

**Published:** 2026-01-14

**Authors:** Wei Zhang, Yemei Dai, Wei Zhang, Yuze Wang, Lihua Wang

**Affiliations:** 1 Department of Nephrology, Shanxi Medical University Second Hospital, Taiyuan, China; 2 Shanxi Kidney Disease Institute, Taiyuan, China; 3 Institute of Nephrology, Shanxi Medical University, Taiyuan, China; 4 Department of Nephrology, Changzhi Medical College Affiliated Heji Hospital, Changzhi, China; 5 College of Computer Science and Cyber Security (Oxford Brookes College), Chengdu University of Technology, Chengdu, China

**Keywords:** chronic kidney diseases, glomerulonephritis, immunoglobulin A nephropathy, immunosuppressive therapy, membranous nephropathy, kidney disease progression, minimal change disease, traditional chinese medicine

## Abstract

**Background:**

This study aims to investigate patients with the three major types of primary glomerular diseases who underwent kidney biopsy at our center, with the objectives of characterizing their clinical phenotypes and pathological features, and identifying risk factors for clinical outcome events.

**Methods:**

Between January 2013 and December 2023, consecutive patients diagnosed with membranous nephropathy (MN), immunoglobulin A nephropathy (IgAN), and minimal change disease (MCD) by kidney biopsy were included in this retrospective follow-up study. Outcome measures included proteinuria remission and kidney disease progression events. Multivariate-adjusted Cox proportional hazards models were utilized.

**Results:**

A total of 608 patients were included in the follow-up cohort, comprising 438 with MN, 110 with IgAN, and 60 with MCD. Clinical remission was achieved in 481 (79.1%) patients, including 333 (54.8%) with complete remission (CR) and 148 (24.3%) with partial remission (PR). Kidney disease progression occurred in 79 (13.0%) patients. After balancing for baseline data and pathological diagnoses in relation to different outcomes, 24-h urinary total protein (24 h-UTP; ≥ 3.5 g/d vs. < 3.5 g/d: HR 1.35, 95% CI 1.10–1.64, *p* = 0.003), low-density lipoprotein (LDL; HR 0.91, 95% CI 0.86–0.96, *p* < 0.001), pathological diagnoses (MN vs. MCD: HR 0.68, 95% CI 0.50–0.92, *p* = 0.011), and interstitial fibrosis and tubular atrophy (IFTA) were significantly associated with proteinuria remission. History of hypertension (HR 2.37, 95% CI 1.32–4.25, *p* = 0.004), and the presence of nodular mesangial sclerosis (HR 1.79, 95% CI 1.01–3.16, *p* = 0.045) were identified as independent risk factors for kidney disease progression. A significant interaction was observed between disease duration and pathological diagnoses. Subgroup analysis indicated that longer disease duration was an independent risk factor for kidney disease progression in patients with MN (HR 1.04, 95% CI 1.01–1.07, *p* = 0.013).

**Conclusion:**

Undertaken at a single center, this study outlines the spectrum of current treatments, clinical outcomes, and factors influencing these outcomes among patients newly diagnosed with the three principal glomerular diseases through kidney biopsy.

## Introduction

Primary glomerulonephritis (PGN) is the most common kidney disease (1) and a leading cause of end-stage kidney disease (ESKD) (2, 3). A notable decline was observed in the proportion of incident hemodialysis cases caused by glomerulonephritis (GN), falling from 51.3% in 2012 to 28.7% in 2022; nevertheless, GN persisted as the second most common etiology of ESKD (2). Distinct regional variations in the manifestations of glomerular diseases are observed. The incidence of immunoglobulin A nephropathy (IgAN) is significantly higher in Asia (4, 5), whereas the spectrum in North America is characterized by a greater prevalence of focal segmental glomerulosclerosis (FSGS) (3).

Analysis of kidney biopsy data from Chinese patients revealed that PGN continues to be the leading form of kidney disease. The detection rate of PGN among kidney biopsy patients was 66.92–71.10% (1, 6, 7). Among the various pathological diagnoses, IgAN showed the highest prevalence (42.30–48.88%), exceeding that of MN (20.67–28.55%), mesangial proliferative glomerulonephritis (MsPGN, 11.30%), minimal change disease (MCD; 5.20–13.30%), and FSGS (6.50–16.79%). Compared to historical data, contemporary studies demonstrate a declining detection rate of both IgAN and MsPGN. This epidemiological transition mirrors the evolving landscape of glomerular diseases (characterized by increased MN prevalence) while simultaneously reflecting diagnostic advancements in distinguishing mesangial proliferative variants.

In the management of glomerular diseases, early identification of potential indicators of disease progression contributes to precision in diagnosis and treatment, delays disease progression, and improves prognosis. The assessment of chronic kidney disease (CKD) is generally based on three key factors: etiology, estimated glomerular filtration rate (eGFR), and urinary albumin level. Accordingly, a rational testing strategy should be adopted: in addition to the urine protein-to-creatinine ratio (uPCR), 24-h urinary total protein (24 h-UTP) quantification should also be considered an essential measure (8). When evaluating the progression of renal function, eGFR formulas based on cystatin C are more sensitive than traditional creatinine-based formulas and can serve as an early detection tool for renal function decline (9). This approach should be combined with the measurement of validated novel biomarkers to enhance the effectiveness of early screening and dynamic monitoring (10–12). In terms of treatment, lifestyle modifications—including smoking cessation, salt restriction, weight reduction (13), and blood pressure control—should form the cornerstone, supplemented by an integrated therapeutic approach that combines Western medicine with multi-target traditional Chinese medicine (TCM), along with continuous monitoring of relevant clinical indicators.

General therapeutic principles for glomerular diseases integrate core supportive care with precise immunosuppression (14). The cornerstone of supportive care is the initiation of an angiotensin-converting enzyme inhibitor (ACEI) or angiotensin II receptor blocker (ARB) in all patients, which should be titrated to the maximum tolerated dose to achieve a systolic blood pressure under 120 mmHg and a proteinuria level below 1 g/day, acknowledging that specific targets vary by disease. For immunosuppression, the objectives are to prevent acute injury, halt disease progression, and minimize toxicity. The standard armamentarium includes glucocorticoids, calcineurin inhibitors (CNIs), mycophenolate mofetil (MMF), rituximab (RTX), and cyclophosphamide (CTX). Critical safety protocols require pre-treatment screening for latent infections, ongoing drug concentration monitoring, and concomitant prophylactic strategies for bone and gastrointestinal protection, alongside fertility preservation as indicated.

Recent years have seen advances in research on TCM compounds for the treatment of glomerular diseases through multi-pathway mechanisms. For example, Dioscorea nipponica Makino has been shown to exert therapeutic effects in chronic kidney disease (CKD) by modulating pathways such as oxidative stress and inhibiting fibrosis (15). Meanwhile, other TCM agents and formulas—including Astragalus membranaceus, Tripterygium wilfordii, curcumin, and Zhenwu Decoction—focus more on repairing podocyte injury (16). Together, these studies illustrate the multi-target and multi-pathway characteristics of TCM interventions. However, there remains a lack of large-scale clinical trials and systematic toxicity data. Future research should further clarify their mechanisms of action and establish standardized clinical protocols.

The Nephrotic Syndrome Study Network (NEPTUNE) investigation revealed significant associations between clinical characteristics and complete proteinuria remission in patients with primary glomerular diseases. Key predictive factors included baseline proteinuria levels prior to kidney biopsy, histopathological classification, and immunosuppressive treatment regimens (17). Systematic analyses have been conducted regarding renal outcomes, including progression to ESKD or mortality, across different histological types of primary glomerulopathie (18–20). Nevertheless, comprehensive investigations into proteinuria remission patterns and their impact on renal function progression in Chinese patients with primary glomerular diseases remain substantially underexplored.

Utilizing a retrospective cohort design, this study investigates a single-center population with kidney biopsy-confirmed primary glomerular disease. By analyzing comprehensive clinical baseline and follow-up data, it seeks to elucidate the factors predictive of clinical proteinuria remission and kidney disease progression.

## Methods

### Study patients and follow-up cohort

This single-center retrospective study enrolled all consecutive patients who underwent kidney biopsy and were diagnosed with PGN at Changzhi Medical College Affiliated Heji Hospital, between January 2013 and December 2023. The indications for kidney biopsy are determined by clinicians based on disease characteristics and related guideline, and each patient has signed an informed consent form for kidney biopsy as well as a consent form for clinical follow-up. Renal pathological specimens were evaluated by KingMed Diagnostics, Guangzhou. Based on kidney biopsy findings, patients were categorized into the following groups: MN, IgAN, MCD, and other primary glomerulonephritides. Clinical diagnosis data were extracted from medical records coded according to the International Classification of Diseases, 10th Revision (ICD-10).

We retrieved patients diagnosed with GN who underwent kidney biopsy from our hospital’s medical record system and documented their pathological diagnoses and age at disease onset. The most recent laboratory results and demographic data prior to kidney biopsy were recorded as baseline characteristics. For patients enrolled in the cohort and regularly followed up at our hospital, follow-up data were collected at 1–3 month intervals for at least 1 year, with all follow-up measurements recorded. The final date for data collection was from December 20 to 31, 2024, during which all enrolled patients were subjected to follow-up and outcome confirmation, either on-site or through telephone. Additionally, every 3 months, we assessed and confirmed the following outcomes: proteinuria remission status, changes in eGFR, survival status (including death and progression to ESKD). We included only those patients for whom complete baseline and follow-up data were available.

This study was approved by the Ethics Committee of Heji Hospital Affiliated to Changzhi Medical College (Approval No. 25–074) and was conducted in strict accordance with the principles of the Declaration of Helsinki. Informed consent for both clinical data collection and the kidney biopsy procedure was obtained as a single document prior to the kidney biopsy.

In line with the current Kidney Disease: Improving Global Outcomes (KDIGO) guidelines (14), MN is categorized into low, medium, high, and very high-risk groups according to 24 h-UTP and eGFR levels. For low-risk patients, follow-up observation or renin-angiotensin system inhibitors (RASi) therapy is recommended. Treatment for medium, high, and very high-risk patients involves corticosteroids and/or immunosuppressive/biologic agents, with the specific regimen determined by the treating clinician.

Following the established therapeutic protocols, enrolled patients were categorized into treatment and non-treatment groups. The treatment group was further divided into: RASi therapy and immunosuppressive therapy (IST). IST regimens consisted of glucocorticoids, immunosuppressive drugs, or targeted biologics, used either alone or in any combination. Specifically, immunosuppressive agents included CTX, CNIs (cyclosporine A, tacrolimus), hydroxychloroquine, tripterygium glycosides, and MMF, while rituximab (RTX) was the only biologic agent utilized within the scope of this study.

### Clinical outcomes

In this longitudinal assessment, we evaluated two primary endpoints: proteinuria response and kidney progression events. Proteinuria outcomes were classified as follows: (1) Complete remission (CR): CR was characterized by a decrease in 24 h-UTP excretion to ≤ 0.3 g/day, along with normalized serum albumin levels and stable renal function; (2) Partial remission (PR): PR was defined as ≥ 50% reduction in the 24 h-UTP ratio and a final excretion of ≤ 3.5 g/day; (3) Non-response (NR): NR was defined as 24 h-UTP remaining above 3.5 g/ day throughout follow-up, with a urinary protein reduction of less than 50%; (4) Relapse: Relapse was defined as new development of the 24 h-UTP > 3.5 g/d after CR or PR. Remission of proteinuria encompassed both CR and PR.

Kidney disease progression event was characterized by either: (i) ≥ 30% decline in eGFR over 24 months, or (ii) ESKD persisting ≥ 4 weeks (21). ESKD criteria included: commencement of maintenance dialysis, renal transplantation, or sustained eGFR < 15 mL/min/1.73 m^2^. The CKD-EPI creatinine-based formula was employed for GFR estimation (22).

### Statistical analysis

Categorical data were presented as number (percentage) and compared using the χ^2^ test or Fisher’s exact test. Continuous data were reported as mean ± standard deviation or median (interquartile) and compared using the t test, one-way analysis of variance (ANOVA), or Kruskal-Wallis test.

Initially, univariate Cox proportional hazards regression was employed to screen potential predictors for the two defined events: proteinuria remission and kidney disease progression (coded as 1 for event occurrence, 0 for censoring). Subsequently, all clinically relevant variables that demonstrated statistical significance (*p* < 0.05) in the univariate analysis were deemed eligible for inclusion in the multivariate Cox models to obtain adjusted hazard ratios. The observation period spanned from the date of kidney biopsy to the occurrence of primary endpoints.

To account for heterogeneity among pathological diagnoses (MN, IgAN, MCD), we used an integrated approach: pathological diagnoses (as dummy variables) and all covariates were included in a multivariable Cox model in the overall cohort, with systematic testing of prespecified interactions between key variables and diagnoses. Significant interactions (*p* < 0.05) led to separate hazard ratio reporting by pathological subtype. Subgroup analyses by pathological diagnosis were also performed for a more complete view.

Time-to-event outcomes were visualized using Kaplan–Meier survival curves, with between-group differences evaluated by log-rank testing. All statistical tests were two-tailed, with *α* = 0.05 serving as the threshold for statistical significance. Data processing and statistical analyses were conducted using IBM SPSS Statistics (version 26.0; Armonk, NY, United States) and R (version 4.4.2).

## Results

### Study cohort

**Figure 1:**
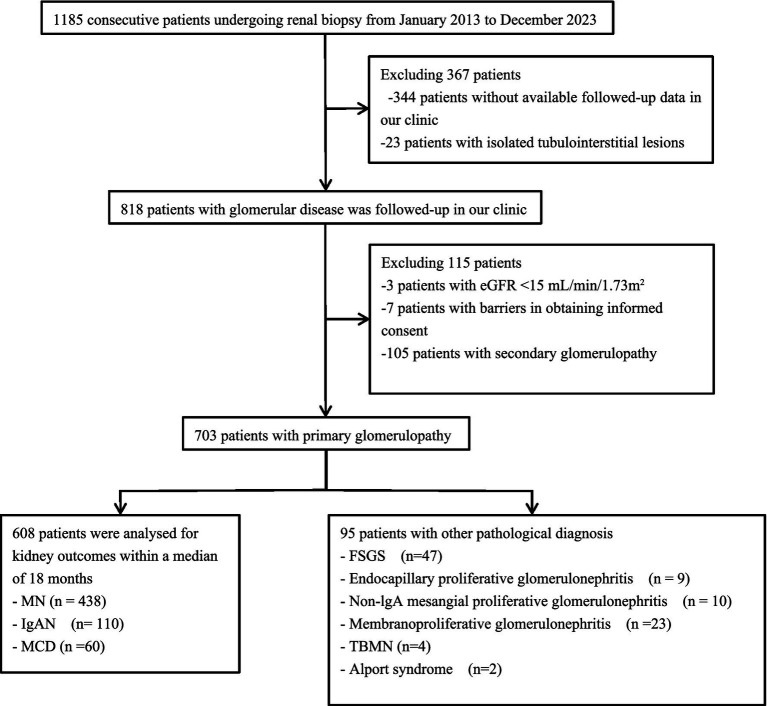
Flowchart of patient selection. eGFR, estimated glomerular filtration rate; FSGS, focal segmental glomerulosclerosis; IgAN, immunoglobulin A nephropathy; IgG4, immunoglobulin G4; MN, membranous nephropathy; MCD, minimal change disease; TBMN, thin basement membrane nephropathy.

Between January 2013 and December 2023, a total of 1,185 consecutive patients underwent kidney biopsy. After excluding 344 patients who were lost to follow-up and 23 cases diagnosed with tubulointerstitial nephropathy, 818 patients were ultimately included in the follow-up study (Figure 1; Supplementary Table S1). Among these, 703 cases were diagnosed with primary glomerulopathy (PGN). The top three diseases within this category were MN (438 cases), IgAN (110 cases), and MCD (60 cases). Among the 95 patients with other types of PGN, FSGS was the most common, accounting for 47 cases in total (Figure 1).

Baseline characteristics were compared across different kidney pathological classifications, with detailed results presented in Table 1. In this study cohort, the mean age at kidney biopsy was 47 years, with 60.0% (422/703) of patients being male. Clinically, 67.6% (475/703) of biopsied patients presented with nephrotic syndrome (NS). Overall, among the three PGN, MN patients exhibited the oldest age at disease onset and the highest eGFR values, while IgAN patients demonstrated the lowest 24 h-UTP levels and highest serum albumin levels. Patients with MCD showed the highest total cholesterol and the lowest serum albumin levels. Histopathological comparisons revealed that MCD had the lowest incidence of renal arteriolar hyalinosis (10.0%), whereas IgAN demonstrated the highest frequencies of crescent formation (50.9%) and segmental glomerular mesangial sclerosis (52.7%).

**Table 1:** Baseline clinical and histopathologic characteristics in patients with different types of primary glomerulopathy.

Characteristics	Total	MN	IgAN	MCD	Other^a^	*p* value^b^
Patients, n	703	438	110	60	95	
Male sex	422 (60.0)	259 (59.1)	67 (60.9)	37 (61.7)	59 (62.1)	0.94
Age at biopsy, years	47.0 (38.0, 56.0)	50.0 (41.8, 58.0)	38.0 (28.0, 51.0)	43.0 (27.0, 55.0)	44.0 (36.0, 56.0)	**<0.001**
Duration of illness, months	11.0 (9.0, 13.0)	11.0 (9.0, 13.0)	9.0 (8.0, 12.0)	10.0 (8.0, 18.8)	12.0 (9.0, 15.0)	**<0.001**
Follow-up duration	19.0 (14.0, 28.0)	18.0 (14.0, 27.0)	22.0 (16.0, 33.25)	16.5 (9.0, 29.0)	19.0 (14.0, 26.0)	
Family history of kidney disease	18 (2.6)	10 (2.3)	0	0	8 (8.4)	**0.001**
Smoking history	231 (32.9)	141 (32.2)	36 (32.7)	27 (45.0)	27 (28.4)	0.18
Alcohol history	191 (27.2)	117 (26.7)	33 (30.0)	21 (35.0)	20 (21.1)	0.25
Clinical syndromed^c^						**<0.001**
NS	475 (67.6)	346 (79.0)	46 (41.8)	46 (76.7)	37 (38.9)	
AKI with NS	22 (3.1)	4 (0.9)	2 (1.8)	14 (23.3)	2 (2.1)	
AKI without NS	8 (1.1)	2 (0.5)	4 (3.6)	0	2 (2.1)	
Progressive CKD	57 (8.1)	14 (3.2)	10 (9.1)	0	33 (34.7)	
Proteinuria	135 (19.2)	72 (16.4)	42 (38.2)	0	21 (22.1)	
Isolated hematuria	6 (0.9)	0	6 (5.5)	0	0	
Comorbidities						
Hypertension	397 (56.5)	258 (58.9)	55 (50.0)	19 (31.7)	65 (68.4)	**<0.001**
Diabetes mellitus	122 (17.4)	77 (17.6)	14 (12.7)	4 (6.7)	27 (28.4)	**0.002**
Systolic BP, mmHg	144.0 (130.0, 158.0)	146.0 (133.0, 160.0)	138.5 (127.0, 148.3)	133.5 (124.0, 148.0)	144.0 (132.0, 163.0)	**<0.001**
Diastolic BP, mmHg	89.0 (80.0, 97.0)	90.0 (82.0, 99.0)	86.0 (76.8, 94.3)	86.5 (80.0, 91.8)	88.0 (80.0, 99.0)	**<0.001**
BMI, kg/m^2^	25.2 (23.0, 27.9)	25.5 (23.4, 28.1)	26.1 (23.0, 28.3)	25.4 (22.6, 28.4)	23.8 (21.7, 25.1)	**<0.001**
Hemoglobin, g/L	140.0 (125.0, 154.0)	142.0 (126.0, 154.0)	139.0 (120.0, 160.0)	148.5 (134.0, 160.0)	127.0 (108.0, 142.0)	**<0.001**
24-h urine total protein, g/d	3.8 (2.4, 5.1)	4.0 (2.8, 5.3)	2.3 (1.1, 4.2)	4.3 (3.0, 5.5)	3.9 (2.0, 5.1)	**<0.001**
24-h urine total protein, g/d						**<0.001**
<3.5 g/d	311 (44.2)	171 (39.0)	73 (66.4)	22 (36.7)	45 (47.4)	
≥3.5 g/d	392 (55.8)	267 (61.0)	37 (33.6)	38 (63.3)	50 (52.6)	
Scr, μmol/L	73.1 (55.8, 93.8)	66.5 (52.3, 80.1)	88.0 (66.3, 110.9)	79.6 (72.5, 96.0)	99.2 (65.0, 139.1)	**<0.001**
eGFR^d^, mL/min/1.73 m^2^	100.1 (74.2, 119.5)	105.7 (88.2, 122.8)	86.1 (62.1, 116.3)	95.2 (73.5, 114.9)	69.9 (40.1, 106.4)	**<0.001**
Urea, mmol/L	5.0 (4.0, 6.7)	4.6 (3.8, 5.7)	5.6 (4.7, 7.0)	6.3 (4.7, 9.0)	6.8 (4.8, 8.4)	**<0.001**
Uric acid, μmol/L	368.0 (309.7, 429.3)	356.7 (299.5, 424.2)	374.4 (325.1, 424.6)	413.0 (326.7, 520.0)	377.0 (320.8, 440.7)	**0.001**
Total cholesterol, mmol/L	6.1 (4.9, 8.0)	6.6 (5.3, 8.2)	4.9 (4.0, 5.4)	8.6 (5.7, 10.5)	5.2 (3.9, 6.7)	**<0.001**
Triglycerides, mmol/L	2.1 (1.6, 3.0)	2.3 (1.7, 3.4)	1.8 (1.5, 2.4)	2.0 (1.6, 2.5)	1.6 (1.2, 2.4)	**<0.001**
Low-density lipoprotein, mmol/L	3.7 (2.9, 5.1)	4.0 (3.2, 5.4)	3.0 (2.6, 3.6)	5.1 (3.2, 6.6)	3.1 (2.4, 4.1)	**<0.001**
Albumin, g/L	28.2 (22.2, 35.3)	27.5 (22.2, 33.3)	36.7 (31.8, 40.6)	19.3 (17.6, 26.2)	32.1 (25.6, 37.4)	**<0.001**
IFTA^e^						**<0.001**
Absence	182 (26.0)	110 (25.1)	10 (9.1)	34 (56.7)	29 (30.5)	
Mild	458 (65.1)	314 (71.7)	68 (61.8)	26 (43.3)	50 (52.6)	
Moderate	51 (7.3)	12 (2.7)	24 (21.8)	0	15 (15.8)	
Diffuse	11 (1.6)	2 (0.5)	8 (7.3)	0	1 (1.1)	
Arteriolar hyalinosis	187 (26.6)	113 (25.8)	36 (32.7)	6 (10.0)	32 (33.7)	**0.004**
Crescent present	85 (12.1)	15 (3.4)	56 (50.9)	0	14 (14.7)	**<0.001**
Nodular mesangial sclerosis present	178 (25.3)	69 (15.8)	58 (52.7)	0	51 (53.7)	**<0.001**
Glomerular obsolescence^e^						**<0.001**
Absence	484 (68.8)	339 (77.4)	48 (43.6)	52 (86.7)	45 (47.4)	
Mild	169 (24.0)	87 (19.9)	42 (38.2)	8 (13.3)	32 (33.7)	
Moderate	42 (6.0)	8 (1.8)	20 (18.2)	0	14 (14.7)	
Diffuse	8 (1.1)	4 (0.9)	0	0	4 (4.2)	

Statistically significant differences are in bold. Values for categorical variables are given as number (percentage); values for continuous variables, as mean ± standard deviation or median (interquartile range). AKI, acute kidney injury; BMI, body mass index; BP, blood pressure; CKD, chronic kidney disease; eGFR, estimated glomerular filtration rate; IFTA, interstitial fibrosis and tubular atrophy; IgAN, immunoglobulin A nephropathy; MN, membranous nephropathy; MCD, minimal change disease; NS, nephrotic syndrome.

a The other diagnoses included focal segmental glomerulosclerosis, endocapillary proliferative glomerulonephritis, non-IgA mesangial proliferative glomerulonephritis, pauci-immune crescentic glomerulonephritis, membranoproliferative glomerulonephritis, membranoproliferative glomerulonephritis, thin basement membrane nephropathy, Alport syndrome.

b Categorical data were compared using the χ^2^ test or Fisher’s exact test. Continuous data were compared using the *t* test, one-way analysis of variance (ANOVA), or Kruskal-Wallis test.

c We defined clinical syndromes as follows: nephrotic syndrome (NS) as 24-h urine protein ≥3.5 g/day with serum albumin <30 g/L; acute kidney injury (AKI) as either serum creatinine increase ≥0.3 mg/dL (≥26.5 μmol/L) within 48 h, ≥1.5 times increase from baseline within 7 days, or urine output <0.5 mL/kg/h for 6 h; CKD as estimated glomerular filtration rate (eGFR) < 60 mL/min/1.73 m^2^ persisting for ≥3 months.

d GFR was calculated using the Chronic Kidney Disease Epidemiology Collaboration (CKD-EPI) equation.

e IFTA and glomerular obsolescence were graded as absent (≤10% involvement), mild (10–30%), moderate (31–60%), or diffuse (>60%) based on the percentage of affected glomeruli or cortical tubulointerstitial area.

### Therapy

Among 438 patients diagnosed with MN, 97 (22.1%) received RASi alone following kidney biopsy, while 43 (9.8%) were treated with glucocorticoids alone, of whom 16/43 (37.2%) achieved 24 h-UTP < 3.5 g/d (Supplementary Table S2). Twelve patients (2.7%) did not receive any pharmacological treatment, with the remaining patients undergoing either monotherapy with IST or combination therapy with glucocorticoids. Among patients receiving IST either as monotherapy or in combination with glucocorticoids, CNIs (212/438, 48.4%) and CTX (37/438, 8.4%) were the most frequently prescribed regimens, followed by RTX (19/438, 4.3%) and tripterygium Wilfordii Hook F (TwHF, 17/438, 3.9%). MMF (2/438, 0.5%) was the least utilized therapeutic option (Table 2).

**Table 2:** Therapy and outcomes of three main primary glomerulopathy.

Therapy and Outcomes	Total (*n* = 608)	MN (*n* = 438)	IgAN (*n* = 110)	MCD (*n* = 60)
Therapy after biopsy				
RASi alone	133 (21.9)	97 (22.1)	30 (27.3)	6 (10.0)
Steroid alone	127 (20.9)	43 (9.8)	39 (35.5)	45 (75.0)
IST alone/combined steroid	330 (54.3)	286 (65.3)	35 (31.8)	9 (8.3)
Cyclophosphamide	55 (9.0)	37 (8.4)	18 (16.4)	0
Calcineurin inhibitor	231 (38.0)	212 (48.4)	13 (11.8)	6 (10.0)
TwHF	21 (3.5)	17 (3.9)	4 (3.6)	0
Mycophenolate mofetil	4 (0.7)	2 (0.5)	2 (1.8)	0
Hydroxychloroquine	10 (1.6)	9 (2.1)	0	1 (1.7)
Rituximab	21 (3.5)	19 (4.3)	0	2 (3.3)
None	18 (3.0)	12 (2.7)	6 (5.5)	0
Remission of proteinuria^a^				
CR	333 (54.8)	216 (49.3)	63 (57.3)	54 (90.0)
PR	148 (24.3)	106 (24.2)	36 (32.7)	6 (10.0)
NR	101 (16.6)	90 (20.5)	11 (10.0)	0
Relapse	26 (3.7)	26 (5.9)	0	0
Kidney disease progression events^b^	79 (13.0)	61 (13.9)	14 (12.7)	4 (6.7)
ESKD	8 (1.3)	2 (0.5)	4 (3.6)	2 (3.3)
30% reduction of baseline eGFR	71 (11.7)	59 (13.5)	10 (9.1)	2 (3.3)

CR, complete remission; ESKD, end stage kidney disease; eGFR, estimated glomerular filtration rate; TwHF, Tripterygium Wilfordii Hook F; IST, immunosuppressive therapy; IgAN, immunoglobulin A nephropathy; MN, membranous nephropathy; MCD, minimal change disease; NR, nonresponse; PR, partial remission; RASi, renin-angiotensin system inhibitors; steroid, glucocorticosteroid.

a The definition of different outcomes: CR was characterized by a decrease in 24 h-UTP excretion to ≤0.3 g/day, along with normalized serum albumin levels and stable renal function. PR was defined as ≥ 50% reduction in the 24 h-UTP ratio and a final excretion of ≤3.5 g/day. NR was defined as 24 h-UTP remaining above 3.5 g/ day throughout follow-up, with a urinary protein reduction of less than 50%. Relapse was defined as new development of the 24 h-UTP >3.5 g/d after CR or PR. Remission of proteinuria was referred to as CR and PR.

b Kidney disease progression event was defined as a 30% decrease in eGFR during the follow-up period or the development of ESKD by at least 4 weeks. ESKD was defined as initiation of long-term dialysis, kidney transplant, or eGFR<15 mL/min/1.73 m2. GFR was estimated according to the CKD Epidemiology Collaboration creatinine equation.

Among IgAN patients, glucocorticoids monotherapy following kidney biopsy was relatively common (39/110, 35.5%), with CTX (18/110, 16.4%) and CNIs (13/110, 11.8%) being the most frequently used IST. MCD patients showed the highest rate of glucocorticoids monotherapy among the three groups at 75% (45/60), while 9 cases (8.3%) received either IST alone or in combination with glucocorticoids (Table 2).

### Clinical outcomes

#### Remission of proteinuria

Patients were stratified based on proteinuria outcomes, and baseline clinical characteristics were compared across these groups (Table 3). During a median follow-up of 18 months, 481/608 (79.1%) patients achieved proteinuria remission, including 333 patients (54.8%) who attained CR. Conversely, 101 patients (16.6%) exhibited treatment resistance with persistent proteinuria, while 26 patients (4.3%) experienced at least one relapse. Adults with MCD demonstrated significantly higher proteinuria remission rates (100%, 60/60) compared to MN (73.5%, 322/438) and IgAN (90.0%, 99/110) cohorts (*p* < 0.001).

**Table 3:** Baseline clinical and histopathologic characteristics by outcomes of proteinuria.

Characteristics	Remission of proteinuria			Persistent proteinuria			*p* value^a^
	Total	CR	PR	Total	NR	Relapse	
Number of patients, n	481 (79.1)	333 (54.8)	148 (24.3)	127 (20.9)	101 (16.6)	26 (4.3)	
Age at biopsy, years	47.0 (37.0, 55.0)	47.0 (37.0, 55.0)	45.0 (36.0, 54.8)	49.0 (41.0, 59.0)	50.0 (41.5, 60.0)	48.0 (41.0, 57.3)	**0.009**
Male sex	277 (57.6)	179 (53.8)	98 (66.2)	86 (67.7)	64 (63.4)	22 (84.6)	**0.042**
Family history of kidney disease	8 (1.7)	8 (2.4)	0	2 (1.6)	2 (2.0)	0	1.00
Smoking history	159 (33.1)	105 (31.5)	54 (36.5)	45 (35.4)	29 (28.7)	16 (61.5)	0.67
Alcohol history	143 (29.7)	87 (26.1)	56 (37.8)	28 (22.0)	22 (21.8)	6 (23.1)	0.10
Duration of illness, months	10.0 (8.0, 13.0)	10.0 (8.0, 13.0)	10.0 (8.0, 13.0)	11.0 (9.0, 13.0)	11.0 (9.0, 13.0)	11.0 (9.0, 13.0)	**0.007**
Comorbidities							
Hypertension	251 (52.2)	156 (46.8)	95 (64.2)	81 (63.8)	69 (68.3)	12 (46.2)	**0.021**
Diabetes mellitus	74 (15.40)	50 (15.0)	24 (16.20)	21 (16.5)	17 (16.8)	4 (15.4)	0.78
BMI, kg/m^2^	25.2 (23.0, 27.8)	24.9 (22.9, 27.3)	26.1 (24.0, 28.4)	27.1 (23.6, 28.6)	26.9 (23.4, 28.8)	27.2 (25.4, 28.5)	**0.002**
24-h urine total protein, g/d	3.9 (2.5, 5.1)	3.6 (2.3, 4.8)	4.3 (3.4, 5.6)	3.4 (2.3, 5.6)	3.3 (2.1, 4.9)	5.0 (3.4, 7.3)	0.59
24-h urine total protein, g/d							**0.001**
<3.5 g/d	194 (40.3)	156 (46.8)	38 (25.7)	72 (56.7)	64 (63.4)	8 (30.8)	
≥3.5 g/d	287 (59.7)	177 (53.2)	110 (74.3)	55 (43.3)	37 (36.6)	18 (69.2)	
eGFR^b^, mL/min/1.73 m^2^	102.0 (77.8, 121.8)	104.1 (81.0, 123.5)	97.0 (73.7, 116.5)	102.2 (85.8, 116.8)	98.9 (78.0, 112.8)	111.1 (101.3, 124.0)	0.96
Hemoglobin, g/L	142.0 (125.0, 157.0)	142.0 (125.0, 158.0)	142.0 (125.0, 155.0)	145.0 (131.0, 155.0)	143.0 (130.0, 154.0)	153.0 (135.0, 167.3)	0.10
Scr, μmol/L	71.9 (54.1, 88.8)	70.8 (52.7, 85.4)	76.9 (58.0, 95.0)	71.6 (59.6, 85.3)	73.0 (59.7, 90.3)	68.2 (53.2, 73.0)	0.92
Urea, mmol/L	4.8 (3.9, 6.5)	4.8 (4.0, 6.4)	4.9 (3.8, 6.6)	4.8 (3.9, 5.6)	4.9 (3.9, 5.8)	4.7 (4.0, 5.5)	0.39
Uric acid, μmol/L	361.4 (301.3, 428.1)	350.3 (297.5, 423.4)	382.2 (317.7, 435.9)	381.8 (327.3, 428.9)	381.8 (334.7, 428.9)	381.4 (325.9, 427.6)	0.13
Total cholesterol, mmol/L	6.3 (5.0, 8.1)	6.3 (5.0, 8.4)	6.3 (5.0, 7.8)	6.6 (5.2, 8.2)	6.4 (5.1, 8.2)	6.6 (5.2, 7.3)	0.38
Triglycerides, mmol/L	2.1 (1.6, 3.0)	2.1 (1.6, 3.1)	2.0 (1.6, 3.0)	2.3 (1.8, 3.5)	2.3 (1.7, 3.5)	3.0 (1.9, 3.6)	**0.024**
Low-density lipoprotein, mmol/L	3.7 (3.0, 5.1)	3.7 (3.0, 5.2)	3.8 (2.8, 5.1)	4.0 (3.2, 5.7)	3.9 (3.1, 5.5)	4.4 (3.4, 6.4)	0.15
Albumin, g/L	28.9 (22.2, 35.1)	28.7 (21.5, 35.8)	28.9 (22.7, 33.9)	25.0 (21.4, 32.2)	25.7 (21.7, 33.7)	23.6 (19.5, 30.7)	**0.004**
Pathological diagnoses							**<0.001**
MN	322 (66.9)	216 (64.9)	106 (71.6)	116 (91.3)	90 (89.1)	26 (100.0)	
IgAN	99 (20.6)	63 (18.9)	36 (24.3)	11 (8.7)	11 (10.9)	0	
MCD	60 (12.5)	54 (16.2)	6 (4.1)	0	0	0	
IFTA^c^							0.71
Absence	125 (26.0)	94 (28.2)	31 (20.9)	29 (22.8)	24 (23.8)	5 (19.2)	
Mild	320 (66.5)	222 (66.7)	98 (66.2)	88 (69.3)	68 (67.3)	20 (76.9)	
Moderate	27 (5.6)	17 (5.1)	10 (6.8)	9 (7.1)	8 (7.9)	1 (3.8)	
Diffuse	9 (1.9)	0	9 (6.1)	1 (0.8)	1 (1.0)	0	
Arteriolar hyalinosis	123 (25.6)	82 (24.6)	41 (27.7)	32 (25.2)	30 (29.7)	2 (7.7)	1.00
Crescent present	58 (12.1)	34 (10.2)	24 (16.2)	13 (10.2)	11 (10.9)	2 (7.7)	0.64
Nodular mesangial sclerosis present	91 (18.9)	49 (14.7)	42 (28.4)	36 (28.3)	27 (26.7)	9 (34.6)	**0.027**
Glomerular obsolescence^c^							0.14
Absence	341 (70.9)	247 (74.2)	94 (63.5)	98 (77.2)	72 (71.3)	26 (100.0)	
Mild	113 (23.5)	79 (23.7)	34 (23.0)	24 (18.9)	24 (23.8)	0	
Moderate	25 (5.20)	7 (2.1)	18 (12.2)	3 (2.4)	3 (3.0)	0	
Diffuse	2 (0.4)	0	2 (1.4)	2 (1.6)	2 (2.0)	0	

Statistically significant differences are in bold. Values for categorical variables are given as number (percentage); values for continuous variables, as mean ± standard deviation or median (interquartile range). BMI, body mass index; CR, complete remission; eGFR, estimated glomerular filtration rate; IFTA, interstitial fibrosis and tubular atrophy; IgAN, immunoglobulin A nephropathy; MN, membranous glomerulonephritis; MCD, minimal change disease; NR, nonresponse; PR, partial remission.

a Categorical data were compared using the χ^2^ test or Fisher’s exact test. Continuous data were compared using the t test, one-way analysis of variance (ANOVA), or Kruskal-Wallis test.

b GFR was calculated using the Chronic Kidney Disease Epidemiology Collaboration (CKD-EPI) equation.

c IFTA and glomerular obsolescence were graded as absent (≤10% involvement), mild (10–30%), moderate (31–60%), or diffuse (>60%) based on the percentage of affected glomeruli or cortical tubulointerstitial area.

Univariate Cox regression analysis revealed significant associations between proteinuria remission and multiple factors including alcohol history, disease duration, levels of 24 h-UTP, serum creatinine, triglycerides, low-density lipoprotein (LDL) levels, Pathological diagnoses, interstitial fibrosis and tubular atrophy (IFTA), and presence of arteriolar hyalinosis (Table 4). Kaplan–Meier curves were generated to assess proteinuria outcomes based on five clinically relevant univariate factors (Supplementary Figure S1).

**Table 4:** Univariable and multivariable analysis of variables for hazard of proteinuria outcome and kidney disease progression events.

Variables	Proteinuria outcome						Kidney disease progression					
	Univariable analysis			Multivariable analysis			Univariable analysis			Multivariable analysis		
	HR	95%CI	*p* value^a^	HR	95%CI	*p* value^a^	HR	95%CI	*p* value^a^	HR	95%CI	*p* value^a^
Age at biopsy, years	0.99	0.99–1.00	0.05				0.75	0.48–1.19	0.22			
Sex												
Male	**Ref**						Ref					
Female	0.93	0.77–1.11	0.40				1.02	1.00–1.04	0.06			
Family history of kidney disease	1.21	0.60–2.45	0.59				0.05	0.00–244.47	0.49			
Smoking history	1.20	0.99–1.46	0.06				2.14	1.37–3.36	**0.001**	1.53	0.81–2.89	0.14
Alcohol history	1.27	1.04–1.55	**0.018**	1.18	0.96–1.45	0.12	2.40	1.53–3.77	**<0.001**	1.68	0.91–3.13	0.14
Duration of illness, months	1.02	1.00–1.03	**0.024**	1.00	0.98–1.02	0.85	1.07	1.04–1.09	**<0.001**	1.05	1.02–1.08	**0.003**
Comorbidities												
Hypertension	0.98	0.82–1.18	0.85				3.14	1.85–5.32	**<0.001**	2.61	1.48–4.59	**0.004**
Diabetes mellitus	1.18	0.92–1.52	0.19				1.62	0.92–2.85	0.09			
BMI, kg/m^2^	1.01	0.98–1.03	0.70				1.05	0.98–1.11	0.15			
24-h urine total protein, g/d												
<3.5 g/d	**Ref**						Ref					
≥3.5 g/d	1.20	1.00–1.45	**0.048**	1.35	1.10–1.64	**0.003**	1.30	0.83–2.06	0.26			
eGFR^b^, mL/min/1.73 m^2^	1.00	1.00–1.00	0.08				1.00	0.99–1.00	0.15			
Hemoglobin, g/L	1.00	1.00–1.01	0.26				1.00	0.99–1.01	0.45			
Scr, μmol/L	1.00	1.00–1.00	**0.045**	1.00	1.00–1.00	0.53	1.01	1.00–1.01	**<0.001**	1.00	1.00–1.00	0.79
Urea, mmol/L	1.01	1.00–1.02	0.20				1.01	0.98–1.04	0.57			
Uric acid, μmol/L	1.00	1.00–1.00	0.34				1.00	1.00–1.00	0.30			
Total cholesterol, mmol/L	1.00	0.96–1.04	0.92				1.05	0.95–1.15	0.32			
Triglycerides, mmol/L	0.93	0.89–0.98	**0.004**	0.95	0.90–1.00	0.07	1.00	0.91–1.10	1.00			
Low-density lipoprotein, mmol/L	0.95	0.91–0.99	**0.016**	0.91	0.86–0.96	**<0.001**	1.00	0.93–1.07	0.97			
Albumin, g/L	1.00	0.99–1.01	0.76				0.97	0.94–1.00	**0.027**	0.97	0.937–1.00	0.17
Pathological diagnoses			**0.001**			**0.040**			0.30			
MCD	**Ref**						Ref					
MN	0.59	0.45–0.78	**<0.001**	0.68	0.50–0.92	**0.011**	1.78	0.65–490	0.27	1.50	0.51–4.44	0.47
IgAN	0.59	0.43–0.82	**0.001**	0.72	0.49–1.04	0.08	1.24	0.41–3.77	0.71	0.86	0.22–3.34	0.83
IFTA^c^			**<0.001**			**<0.001**			**0.029**			0.08
Absence	**Ref**						Ref					
Mild	0.49	0.40–0.61	**<0.001**	0.47	0.37–0.60	**<0.001**	0.46	0.26–0.80	**0.006**	0.46	0.22–0.97	**0.04**
Moderate	0.42	0.27–0.63	**<0.001**	0.34	0.21–0.54	**<0.001**	0.52	0.20–1.34	0.18	0.49	0.14–1.68	0.25
Diffuse	0.36	0.18–0.71	**0.003**	0.20	0.10–0.42	**<0.001**	0.98	0.36–2.70	0.97	1.18	0.22–6.32	0.85
Arteriolar hyalinosis	1.25	1.02–1.54	**0.035**	1.49	1.18–1.86	**0.001**	1.70	1.04–2.77	**0.033**	1.15	0.65–2.03	0.64
Crescent present	0.79	0.60–1.04	0.10				1.39	0.80–2.42	0.24			
Nodular mesangial sclerosis present	0.83	0.66–1.05	0.12				2.07	1.31–3.28	**0.002**	1.79	1.01–3.16	**0.045**
Glomerular obsolescence^c^			0.76						**0.011**			0.55
Absence	**Ref**						Ref					
Mild	0.92	0.74–1.14	0.44				1.06	0.62–1.81	0.82	1.37	0.74–2.56	0.32
Moderate	1.01	0.67–1.52	0.96				3.04	1.57–5.87	**0.001**	2.00	0.72–5.57	0.18
Diffuse	0.58	0.14–2.32	0.44				0.00	0.00–7.15 × 10^268^	0.97	0.00	0.00–7.05 × 10^233^	0.97

Bold values indicate significant factors for the outcome event. BMI, body mass index; CI, confidence interval; eGFR, estimated glomerular filtration rate; HR, hazard ratio; IFTA, interstitial fibrosis and tubular atrophy; IgAN, immunoglobulin A nephropathy; MN, membranous nephropathy; MCD, minimal change disease; Ref, reference.

a Comparisons of p values in characteristics were performed using the Cox proportional hazard regression model. Parameters that were significant in the univariable analysis (*p* < 0.05) were included in this multivariate.

b GFR was estimated according to the CKDEpidemiology Collaboration creatinine equation.

c IFTA and glomerular obsolescence were graded as absent (≤10% involvement), mild (10–30%), moderate (31–60%), or diffuse (> 60%) based on the percentage of affected glomeruli or cortical tubulointerstitial area.

The nine statistically significant variables identified in univariate analysis were included in the multivariate Cox proportional hazards model. We tested the interaction terms between pathological diagnoses and each of the following variables: 24 h-UTP, LDL, IFTA, and arteriolar hyalinosis. No statistically significant interactions were found (Supplementary Table S3). Accordingly, the hazard ratios for these factors are reported as unified estimates from the overall population model. Results revealed that 24 h-UTP at kidney biopsy (HR 1.35, 95% CI 1.10–1.64, *p* = 0.003), serum LDL cholesterol levels (HR 0.91, 95% CI 0.86–0.96, *p* < 0.001), pathological classification (MN vs. MCD, HR 0.69, 95% CI 0.50–0.92, *p* = 0.011), IFTA severity (Mild vs. absence HR 0.47, 95% CI 0.37–0.59, *p* < 0.001; Moderate vs. absence HR 0.34, 95% CI 0.21–0.54, *p* < 0.001; Severe vs. absence HR 0.20, 95% CI 0.10–0.42, *p* < 0.001), presence of arteriolar hyalinosis (HR 1.49, 95% CI 1.18–1.86, *p* = 0.001) were significantly correlated with remission of proteinuria (Supplementary Figure S2). In subgroup analyses, different therapeutical regimens were associated with proteinuria remission in patients with MN but not in those with IgAN (Supplementary Tables S4, S5).

#### Kidney disease progression

During a median follow-up of 18 months in 608 patients, no deaths were recorded. Kidney disease progression occurred in 79 individuals (13.0%), of whom 8 (1.3%) reached ESKD and 71 (11.7%) experienced an eGFR decline of ≥30% (Table 2). Among the 8 ESKD cases, the underlying diagnoses were MN (*n* = 2), IgAN (*n* = 4), and MCD (*n* = 2). Notably, of the 438 MN patients included in the study, 59 (13.5%) experienced a decline in eGFR exceeding 30%. Comparative analysis of baseline characteristics between patients with and without kidney disease progression is presented in Table 5.

**Table 5:** Baseline clinical and histopathologic characteristics by kidney disease progression events.

Characteristics	With kidney disease progression events (*n* = 79)	Without kidney disease progression events (*n* = 529)	*p* value^a^
Male sex	48 (60.8)	315 (59.5)	0.90
Age at biopsy, years	54.0 (42.0, 61.0)	46.0 (38.0, 55.5)	**0.008**
Duration of illness, months	12.0 (10.0, 15.0)	10.0 (8.0, 13.0)	**<0.001**
Family history of kidney disease	0	10 (1.9)	0.38
Smoking history	36 (44.3)	169 (31.9)	**0.040**
Alcohol history	34 (43.0)	137 (25.9)	**0.002**
Pathological diagnoses			**0.026**
MN	61 (77.2)	377 (71.3)	
IgAN	14 (17.7)	96 (18.1)	
MCD	4 (5.1)	56 (10.6)	
Clinical syndromed^b^			**<0.001**
NS	65 (82.3)	373 (70.5)	
AKI with NS	2 (2.5)	18 (3.4)	
AKI without NS	0	6 (1.1)	
Progressive CKD	6 (7.6)	18 (3.4)	
Proteinuria	6 (7.6)	108 (20.4)	
Isolated hematuria	0	6 (1.1)	
Comorbidities			
Hypertension	60 (75.9)	272 (51.4)	**<0.001**
Diabetes mellitus	15 (19.0)	80 (15.1)	0.41
Systolic BP, mmHg	146.0 (132.0,158.0)	143.0 (130.0, 157.0)	0.51
Diastolic BP, mmHg	88.0 (82.0,99.0)	90.0 (80.0, 97.0)	0.88
BMI, kg/m^2^	26.6 (23.6,28.6)	25.4 (23.3, 28.1)	0.21
Hemoglobin, g/L	135.0 (127.0,149.0)	143.0 (126.0, 158.0)	0.05
24-h urine total protein, g/d	4.2 (3.0,5.7)	3.7 (2.4, 5.1)	0.11
24-h urine total protein, g/d			0.28
<3.5 g/d	30 (38.0)	236 (44.6)	
≥3.5 g/d	49 (62.0)	293 (55.4)	
Scr, μmol/L	68.6 (52.4, 81.2)	72.4 (55.4, 88.6)	0.36
eGFR^c^, mL/min/1.73 m^2^	102.2 (78.3, 122.6)	102.0 (79.2, 120.6)	0.86
Urea, mmol/L	4.7 (4.0, 6.5)	4.8 (3.9, 6.3)	1.00
Uric acid, μmol/L	374.4 (324.4, 436.7)	362.6 (306.4, 428.1)	0.24
Total cholesterol, mmol/L	6.6 (5.2, 8.2)	6.3 (5.0, 8.1)	0.35
Triglycerides, mmol/L	2.7 (1.8, 3.5)	2.1 (1.6, 3.0)	**0.011**
Low-density lipoprotein, mmol/L	4.0 (3.3, 5.7)	3.7 (3.0, 5.1)	**0.025**
Albumin, g/L	27.0 (21.4, 33.3)	28.3 (21.8, 35.2)	0.16
IFTA^d^			**0.015**
Absence	17 (21.5)	137 (25.9)	
Mild	51 (64.6)	357 (67.5)	
Moderate	6 (7.6)	30 (5.7)	
Diffuse	5 (6.3)	5 (0.9)	
Arteriolar hyalinosis	24 (30.4)	131 (24.8)	0.33
Crescent present	16 (20.3)	55 (10.4)	**0.015**
Nodular mesangial sclerosis present	29 (36.7)	98 (18.5)	**<0.001**
Glomerular obsolescence^d^			**0.001**
Absence	48 (60.8)	391 (73.9)	
Mild	20 (25.3)	117 (22.1)	
Moderate	11 (13.9)	17 (3.2)	
Diffuse	0	4 (0.8)	

Statistically significant differences are in bold. Values for categorical variables are given as number (percentage); values for continuous variables, as mean ± standard deviation or median (interquartile range). AKI, acute kidney injury; BMI, body mass index; BP, blood pressure; CKD, chronic kidney disease; eGFR, estimated glomerular filtration rate; IFTA, interstitial fibrosis and tubular atrophy; IgAN, immunoglobulin A nephropathy; MN, membranous nephropathy; MCD, minimal change disease; NS, nephrotic syndrome.

a Categorical data were compared using the χ2 test or Fisher’s exact test. Continuous data were compared using the t test, one-way analysis of variance (ANOVA), or Kruskal-Wallis test.

b We defined clinical syndromes as follows: nephrotic syndrome (NS) as 24-h urine protein ≥3.5 g/day with serum albumin <30 g/L; acute kidney injury (AKI) as either serum creatinine increase ≥0.3 mg/dL (≥26.5 μmol/L) within 48 h, ≥1.5 times increase from baseline within 7 days, or urine output <0.5 mL/kg/h for 6 h; chronic kidney disease (CKD) as estimated glomerular filtration rate (eGFR) < 60 mL/min/1.73 m^2^ persisting for ≥3 months.

c GFR was calculated using the Chronic Kidney Disease Epidemiology Collaboration (CKD-EPI) equation.

d IFTA and glomerular obsolescence were graded as absent (≤10% involvement), mild (10–30%), moderate (31–60%), or diffuse (>60%) based on the percentage of affected glomeruli or cortical tubulointerstitial area.

The univariable Cox regression analysis identified several significant risk factors for kidney disease progression, including alcohol history, smoking history, disease duration, hypertension history, serum creatinine levels, albumin levels, and four histopathological features (IFTA, arteriolar hyalinosis, nodular mesangial sclerosis presence, and glomerular obsolescence; Table 4). Kaplan–Meier curves were constructed to assess kidney disease progression based on seven significant univariate categorical variables (Supplementary Figure S3).

To account for heterogeneity across different pathological diagnoses (MN, IgAN, MCD), pathological diagnoses was included as a variable in the multivariable analysis. All eleven variables that reached statistical significance in univariable analyses were entered into a multivariable Cox proportional hazards model. Independent predictors of kidney disease progression were history of hypertension (HR 2.37, 95 % CI 1.32 - 4.25, *p* = 0.004), and the presence of nodular mesangial sclerosis (HR 1.79, 95 % CI 1.01 - 3.16, *p* = 0.045; Supplementary Figure S4). Interaction analysis suggested that the effect pattern of disease duration on kidney disease progression might differ across pathological diagnoses (interaction *p* = 0.001; Supplementary Table S6). Subgroup analysis stratified by pathological diagnoses revealed that, in patients with MN, longer disease duration was an independent risk factor for kidney disease progression (HR 1.04, 95% CI 1.01–1.07, *p* = 0.013). In contrast, among patients with IgAN, although its effect differed significantly from that in minimal change disease (MCD) (interaction HR 1.16, *p* = 0.035), disease duration did not show a statistically significant independent effect within the IgAN subgroup itself (HR 1.0, 95% CI 0.78–1.31, *p* = 0.96) (Supplementary Figure S5). No significant association was observed between different therapeutic regimens and kidney disease progression events in either the MN or IgAN patient groups (Supplementary Tables S4, S5).

## Discussion

This retrospective single-center study analyzed the disease distribution and clinical outcomes of PGN using kidney biopsy records from 2013 to 2023. After rigorous screening, 703 patients were enrolled, with MN (62.3%), IgAN (15.6%), and MCD (8.5%) constituting the predominant pathological subtypes. All followed patients received protocol-based care under nephrologist supervision. In the MN/IgAN/MCD subgroup (*n* = 608), proteinuria remission was achieved in 481 cases (79.1%), while 79 patients (13.0%) exhibited renal function deterioration. Notably, MN accounted for 77.9% (61/79) of progression cases, this distribution pattern may reflect both the cohort’s high MN prevalence and the typically prolonged progression to ESKD in IgAN patients (18, 23). Key findings demonstrated that: (1) baseline 24 h-UTP was an independent predictive factor for proteinuria remission (HR 1.35, 95% CI 1.10–1.64, *p* = 0.003); (2) hypertension history independently associated with kidney progression (HR 2.61, 95% CI 1.48–4.59, *p* = 0.004); (3) interstitial IFTA severity correlated with both reduced remission probability and accelerated functional decline; and (4) nodular mesangial sclerosis substantially increased progression risk (HR 1.79, 95% CI 1.01–3.16, *p* = 0.045).

Consistent with the KDIGO clinical practice guidelines (14), patients with MN were stratified into four prognostic categories according to proteinuria levels (24 h-UTP) and renal function (eGFR). In this study, 22.1% (97/438) of MN patients received RASi monotherapy, while 75.1% (329/438) were treated with corticosteroids and/or immunosuppressants, either alone or in combination. This practice deviates from the KDIGO guideline (14), which recommends an initial observation period of at least 6 months to monitor for spontaneous remission. First, many patients already exhibit clinical manifestations of kidney disease for up to 6 months or more before laboratory tests confirm proteinuria and hypoalbuminemia. As a result, clinicians often consider spontaneous remission unlikely. Second, many patients develop complications such as thromboembolism even before undergoing kidney biopsy, indicating a necessity for immediate initiation of IST. Additionally, a considerable number of patients present with high anti-PLA2R antibody titers, leading clinicians to anticipate a lower probability of remission. For these reasons, many patients end up receiving glucocorticoids and/or IST even when they do not strictly meet the indications outlined in the KDIGO guidelines. The deviation from guideline-recommended treatment for MN, as described above, was also evident in the Cure Glomerulonephropathy (CureGN) cohort study (24). In that study, 55% of adults initiated IST within 6 months after kidney biopsy, including patients with a urine protein-to-creatinine ratio below 4 g/g.

Recent decades have witnessed substantial therapeutic advances in IgAN, encompassing endothelin receptor antagonists (ERA) (25, 26), mucosal-targeted corticosteroids (27), complement inhibitors (28–31), and immunomodulators targeting the BLyS/BAFF-APRIL axis (32). According to the 2025 KDIGO guidelines (33), the management of IgAN centers on a proteinuria target of < 0.5 g/day (ideally < 0.3 g/day) and a “dual synergistic” strategy addressing both immunologic drivers and CKD progression. All patients require foundational care: lifestyle adjustments (smoking cessation, weight control, sodium < 2 g/d, exercise) and stringent blood pressure control (≤120/70 mmHg). For patients with risk of progressive kidney function loss, immunologic intervention is advised, preferentially with a 9-month course of Nefecon. Where Nefecon is unavailable, a reduced-dose corticosteroid regimen (e.g., methylprednisolone) with antimicrobial prophylaxis serves as an alternative (33). Notably, antiplatelet agents and azathioprine are discouraged, though mycophenolate mofetil or hydroxychloroquine are corticosteroid-sparing options exclusive to Chinese patients. To counter CKD progression, optimized RASi therapy is essential for all eligible individuals. High-risk patients in approved regions should also receive sparsentan (contraindicated with RASi) and an SGLT2i. Given the marked heterogeneity in disease progression and outcomes, personalized treatment strategies are imperative.

Oral glucocorticoids represent the cornerstone of first-line therapy for adult MCD (34), supported by two randomized controlled trials and extensive observational studies demonstrating remission rates exceeding 80% (35, 36). Treatment regimens can be individualized based on clinical response and patient-specific factors. In this study, 75% (45/60) of MCD patients received glucocorticoid monotherapy. Among these patients, 88.9% (40/45) achieved CR and 11.1% (5/45) attained PR. While the efficacy of steroid therapy in this cohort appears higher than in prior reports, this finding should be interpreted with caution as it may reflect the constraints of our modest sample size rather than a true therapeutic advantage. For patients with relative contraindications to glucocorticoids or those who are unwilling to use glucocorticoids (15% in MCD patients of our cohort), alternative initial agents include CTX, CNIs, RTX or MMF. Relapse is very common in patients with MCD, although it was not observed in the follow-up of this cohort. This may be related to the short follow-up duration and small sample size.

IgAN and MN are the two most prevalent forms of PGN. Globally, the incidence of IgAN exhibits significant geographical variation. In China, it accounts for approximately 39.73–44.4% of PGN cases and is a leading cause of ESKD, whereas MN is a major etiology of antibody-associated NS in adults (1, 37). Recent research has focused on common pathogenic mechanisms underlying both diseases, such as oxidative stress (38), inflammatory fibrosis (39), and dysregulation of signaling pathways (40–43). In terms of intervention, TCM (44), small-molecule inhibitors (38, 42, 43), and certain chemical agents (39) have demonstrated therapeutic potential. Furthermore, relevant signaling pathways (40) and their regulatory molecules (44) may serve as novel biomarkers for disease diagnosis and prognosis evaluation, offering promising directions for future treatment strategies.

A report from the NEPTUNE study showed that among 441 enrolled patients, 46% achieved CR of proteinuria, including 75% with MCD, 34% with FSGS, 31% with MN, and 41% with other pathological diagnoses (17). In the present study, 54.8% (333/608) of the cohort achieved CR. Specifically, the CR rates were 49.3% for MN, 57.3% for IgAN, and 90.0% for MCD, respectively. Notably, a subset of patients with MN in our cohort received glucocorticoids and/or immunosuppressants early in the disease course, which deviates from guideline recommendations. This observation may partially explain why the CR rate in the present study is higher than that reported in previous literature (45, 46). Furthermore, in our subgroup multivariate analysis, we also observed that the choice of treatment regimen was associated with the remission of proteinuria.

Regarding risk factors influencing proteinuria remission, a prospective study from NEPTUNE found that the amount of proteinuria before kidney biopsy, pathological diagnosis, and the use of immunosuppressive agents were all associated with CR. Specifically, patients with higher pre-biopsy proteinuria had 0.3 times the probability of achieving remission compared to those with low proteinuria (17). This study observed that patients with higher baseline proteinuria levels (24 h-UTP ≥ 3.5 g/d) actually had a greater probability of achieving proteinuria remission. This phenomenon may be explained by two factors. First, the study cohort might have included a higher proportion of pathological diagnoses that are highly responsive to treatment and often present with heavy proteinuria, such as MCD or early-stage MN, thereby creating the appearance that higher baseline proteinuria is associated with a higher remission rate. Second, in survival analysis with “proteinuria remission” as the endpoint, patients with more active baseline disease have greater relative potential for clinical improvement, which can lead to a statistically stronger positive association.

Dyslipidemia is highly prevalent among patients with kidney disease. Previous research has indicated that dyslipidemia has a causal relationship with glomerular injury, ultimately leading to glomerulosclerosis (47). Elevated triglycerides (TGs) and reduced high-density lipoprotein cholesterol (HDL-C) levels appear to be independent risk factors for the development of advanced CKD (48). The present results demonstrate that while baseline LDL levels did not differ significantly between patients with and without proteinuria remission (*p* = 0.152), COX regression analysis indicated that, for each 1 mmol/L increase in baseline LDL levels, the probability of achieving proteinuria remission was reduced by a factor of 0.91 (HR 0.91, 95% CI: 0.86–0.96, *p* < 0.001). When stratified by the presence of kidney disease progression, the group with progression exhibited elevated levels of both triglycerides (*p* = 0.011) and LDL (*p* = 0.025) compared to the non-progression group. This observation is supported by a biological rationale: substantial experimental evidence indicates that LDL aggravates renal injury by inducing glomerular podocyte damage, mesangial matrix proliferation, inflammatory responses, and fibrosis, which collectively hinder proteinuria remission (47). Thus, our study corroborates, from a clinical epidemiological perspective, the critical role of LDL management in the context of proteinuria treatment.

The histopathological changes found in kidney biopsy specimens are vital not only for diagnosing kidney diseases but also for assessing prognosis and predicting disease progression. Key histological markers of irreversible renal damage comprise IFTA and glomerulosclerosis. Among these, IFTA is the strongest morphological predictor of CKD progression and prognosis, irrespective of the underlying etiology (49). Evidence from previous studies indicates that, even after accounting for clinical predictors (e.g., eGFR, proteinuria, and clinicopathologic diagnosis), both IFTA and vascular lesions (arterial/arteriolar sclerosis and global glomerulosclerosis) remained independent predictors of kidney disease progression (50). This study incorporated histopathological features of patients to analyze risk factors for proteinuria remission and disease progression in MN, IgAN and MCD. The analysis identified IFTA and arteriolar hyalinosis as risk factors for impaired proteinuria remission, while nodular mesangial sclerosis was associated with kidney disease progression. A significant difference in IFTA was observed between patients with and without kidney disease progression. This is largely consistent with previous findings.

This study has several limitations. First, the decision to perform a kidney biopsy depends on the patient’s clinical manifestations, the clinician’s judgment, and the patient’s consent. Therefore, patients with mild disease or some high-risk patients concerned about biopsy-related risks may decline the procedure. These real-world clinical scenarios can introduce selection bias and unavoidable confounding factors. Second, the potential influence of heterogeneity in clinical management and treatment decisions among different physicians was not accounted for in this study. Third, a key limitation of this study is the potential for selection bias, as we only included patients with complete follow-up data. This likely excluded those lost to follow-up who may have had worse outcomes, possibly overestimating the positive results in our cohort. Additionally, as a retrospective observational study, our outcome analysis incorporated follow-up laboratory results from multiple hospitals, which could introduce detection bias and inter-center variability. Future efforts should therefore focus on building more robust kidney biopsy databases to ensure more complete follow-up tracking and standardized data collection across centers. Fourth, the follow-up duration constrains a full assessment of glomerular disease progression. Extended longitudinal observation is essential to clarify the long-term trajectories of proteinuria and resultant renal outcomes.

In conclusion, our study presents the treatment status, clinical outcomes, and associated influencing factors of the three major types of primary glomerular diseases diagnosed via kidney biopsy. The insights gained from this single-center cohort are directly applicable to and can help inform real-world clinical decision-making.

## Data Availability

The datasets presented in this article are not readily available because data from this study may contain potentially or sensitive patient information. Requests to access the datasets should be directed to wanglh2303@163.com.
