# Supplementary material for: A retrospective single-center cohort study of major subtypes of primary glomerular diseases (MN, IgAN, and MCD): clinical characteristics, prognostic outcomes, and risk factors
Source: Front Med (Lausanne). 2026 Jan 14;12:1741853. doi: 10.3389/fmed.2025.1741853 (PMC12846976; doi:10.3389/fmed.2025.1741853)
Supplement: Supplementary file 1 [file Data_Sheet_1.pdf]

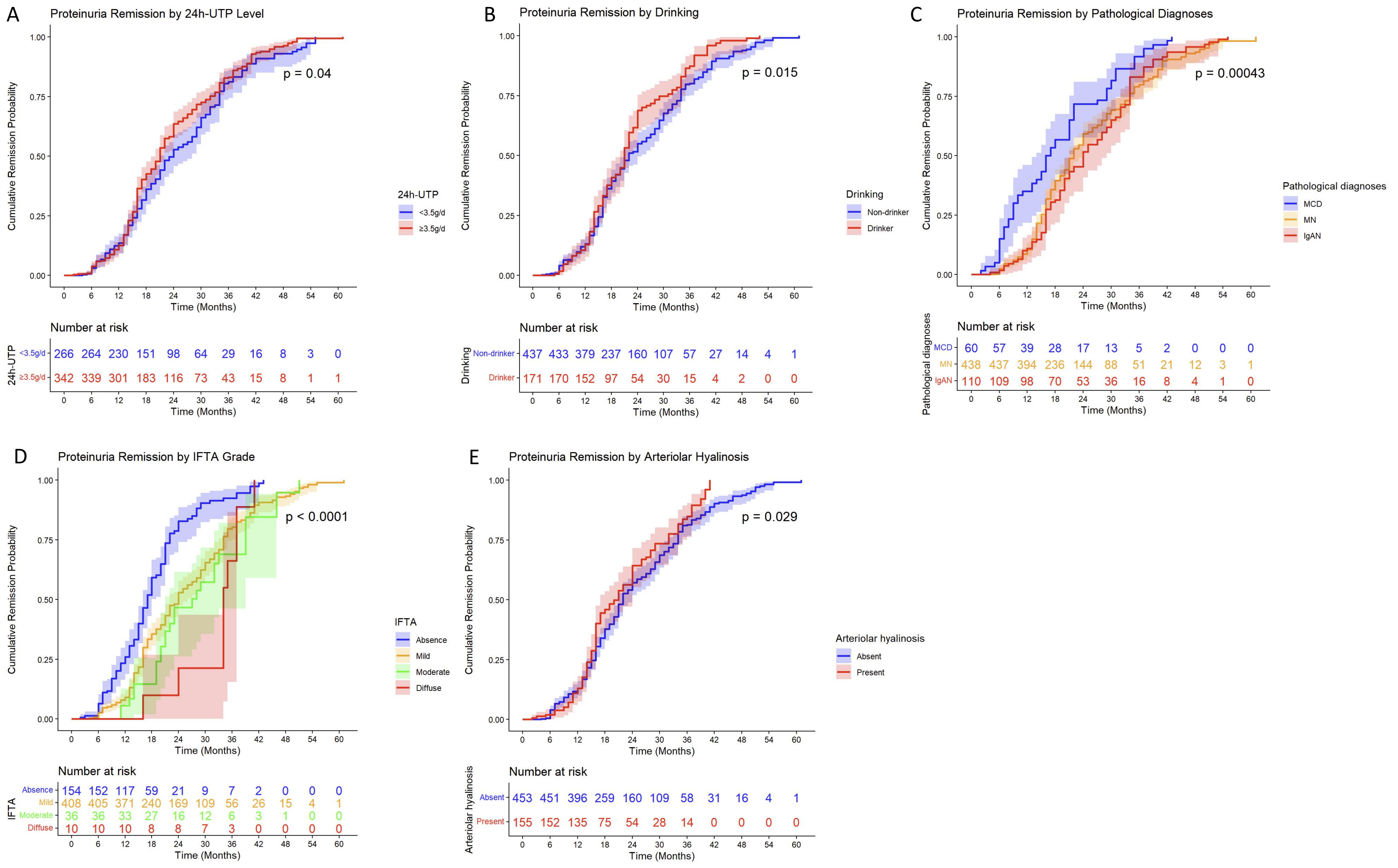

Figure S1 Kaplan-Meier Curves Depicting Proteinuria Remission in Different Subgroups：(A) By Baseline 24-h Urine Total Protein; (B) By History of Alcohol Consumption; (C) By Pathological Diagnosis; (D) By IFTA Grade; (E) By Presence of Arteriolar Hyalinosis

IFTA, interstitial fibrosis and tubular atrophy; IgAN, immunoglobulin A nephropathy; MN, membranous glomerulonephritis; MCD, minimal change disease; UTP, urine total protein

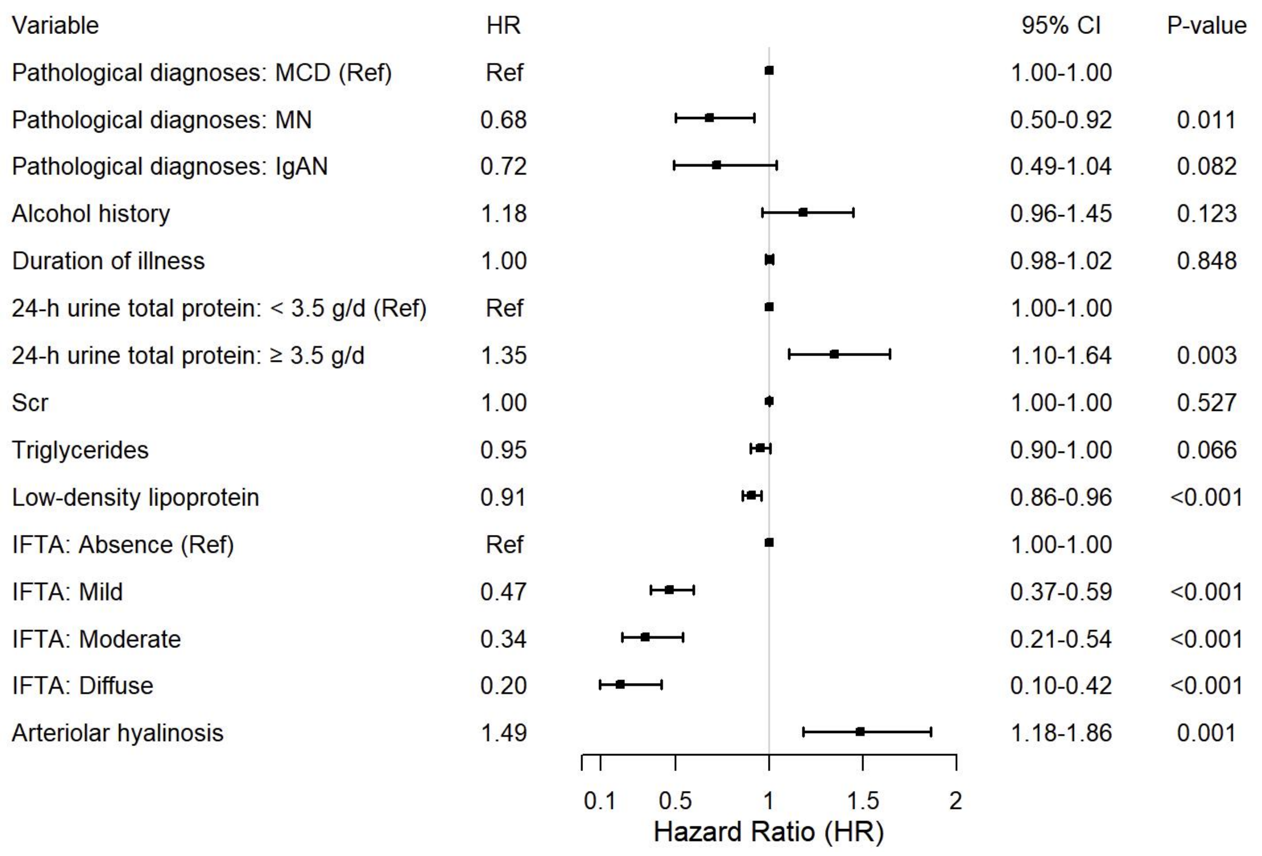

Figure S2 Forest Plot of Multivariable Cox Regression Analysis for Independent Risk Factors of Proteinuria Remission

CI, confidence interval; HR, hazard ratio; IFTA, interstitial fibrosis and tubular atrophy; IgAN, immunoglobulin A nephropathy; MN, membranous nephropathy; MCD, minimal change disease; Ref, reference

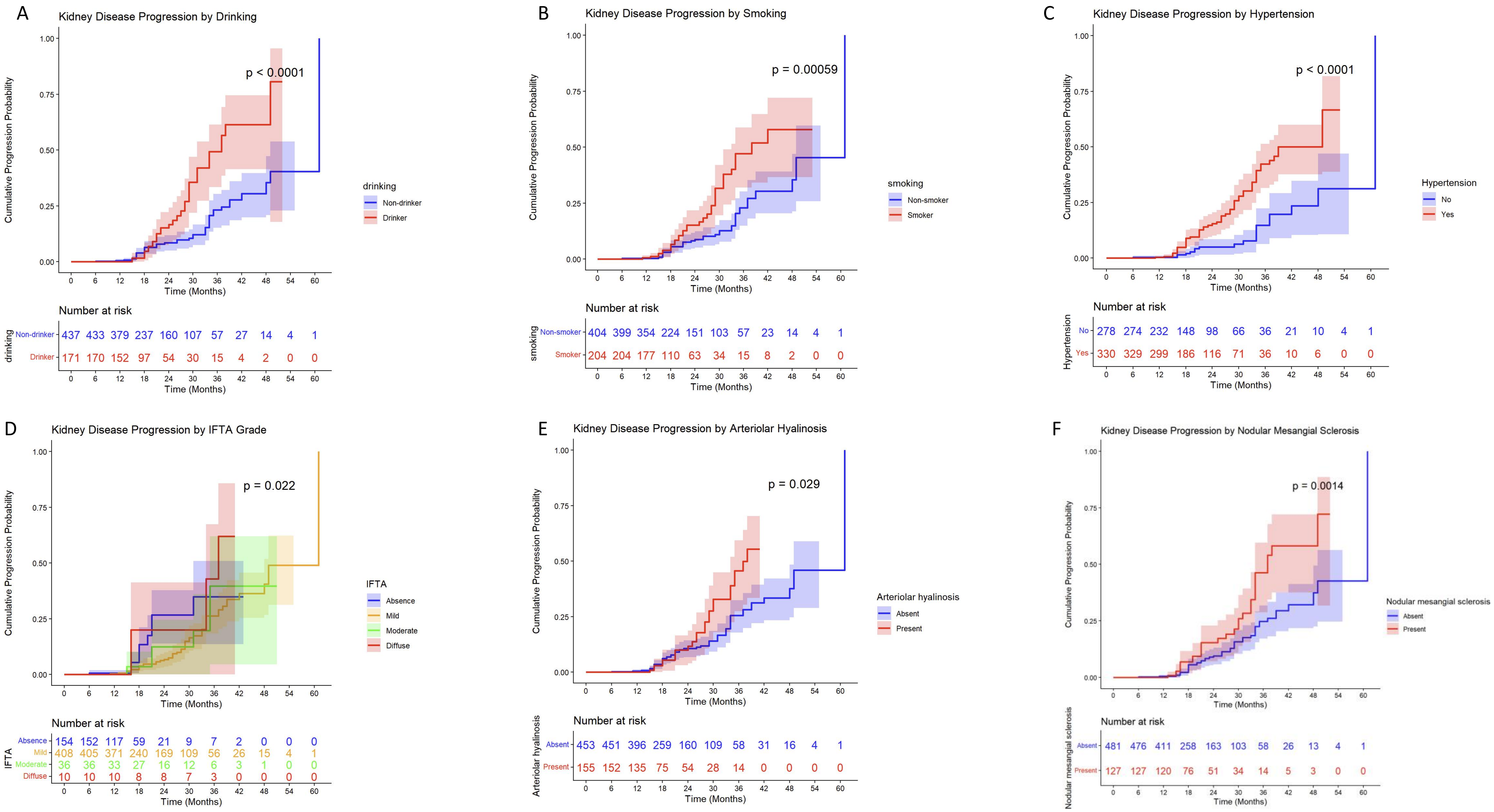
A
Kidney Disease Progression by Drinking
p < 0.0001
Cumulative Progression Probability
Time (Months)
drinking
Non-drinker
Drinker
Number at risk
Non-drinker 437 433 379 237 160 107 57 27 14 4 1
Drinker 171 170 152 97 54 30 15 4 2 0 0
B
Kidney Disease Progression by Smoking
p = 0.00059
smoking
Non-smoker
Smoker
Number at risk
Non-smoker 404 399 354 224 151 103 57 23 14 4 1
Smoker 204 204 177 110 63 34 15 8 2 0 0
C
Kidney Disease Progression by Hypertension
p < 0.0001
Hypertension
No
Yes
Number at risk
No 278 274 232 148 98 66 36 21 10 4 1
Yes 330 329 299 186 116 71 36 10 6 0 0
D
Kidney Disease Progression by IFTA Grade
p = 0.022
IFTA
Absence
Mild
Moderate
Diffuse
Number at risk
Absence 154 152 117 59 21 9 7 2 0 0 0
Mild 408 405 371 240 169 109 56 26 15 4 1
Moderate 36 36 33 27 16 12 6 3 1 0 0
Diffuse 10 10 10 8 8 7 3 0 0 0 0
E
Kidney Disease Progression by Arteriolar Hyalinosis
p = 0.029
Arteriolar hyalinosis
Absent
Present
Number at risk
Absent 453 451 396 259 160 109 58 31 16 4 1
Present 155 152 135 75 54 28 14 0 0 0 0
F
Kidney Disease Progression by Nodular Mesangial Sclerosis
p = 0.0014
Nodular mesangial sclerosis
Absent
Present
Number at risk
Absent 481 476 411 258 163 103 58 26 13 4 1
Present 127 127 120 76 51 34 14 5 3 0 0

G

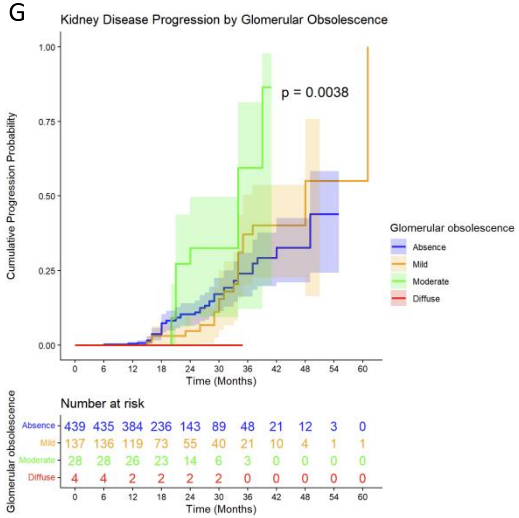

Figure S3 Kaplan-Meier Curves Depicting Kidney Disease Progression in Different Subgroups：(A) By History of Alcohol Consumption (B) By History of Smoking (C) By Comorbid Hypertension (D) By IFTA Grade (E) By Presence of Arteriolar Hyalinosis (F) By Presence of Nodular Mesangial Sclerosis (G) By Degree of Glomerular Obsolescence

IFTA, interstitial fibrosis and tubular atrophy

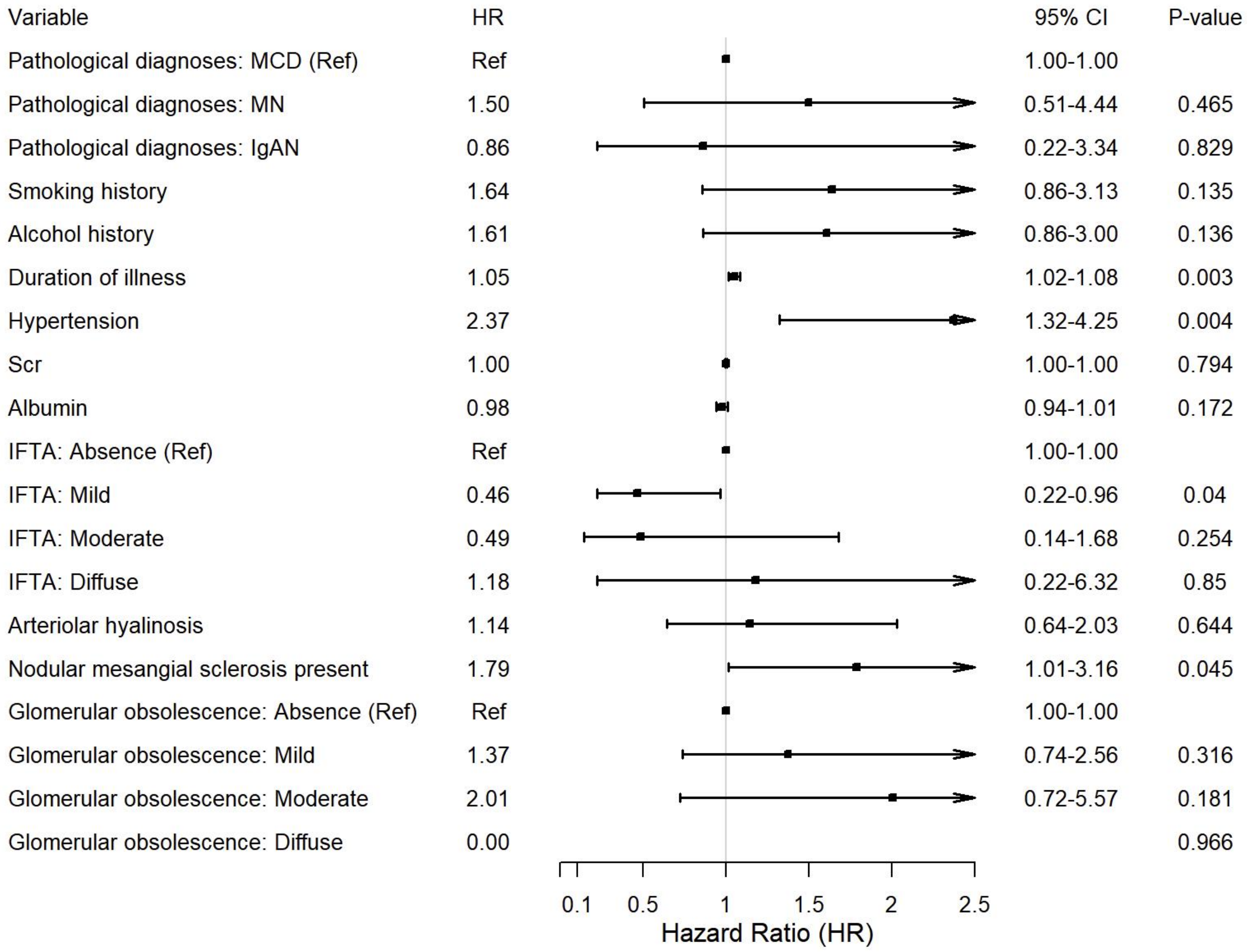

Figure S4 Forest Plot of Multivariable Cox Regression Analysis for Independent Risk Factors of Kidney Disease Progression

CI, confidence interval; HR, hazard ratio; IFTA, interstitial fibrosis and tubular atrophy; IgAN, immunoglobulin A nephropathy; MN, membranous nephropathy; MCD, minimal change disease; Ref, reference

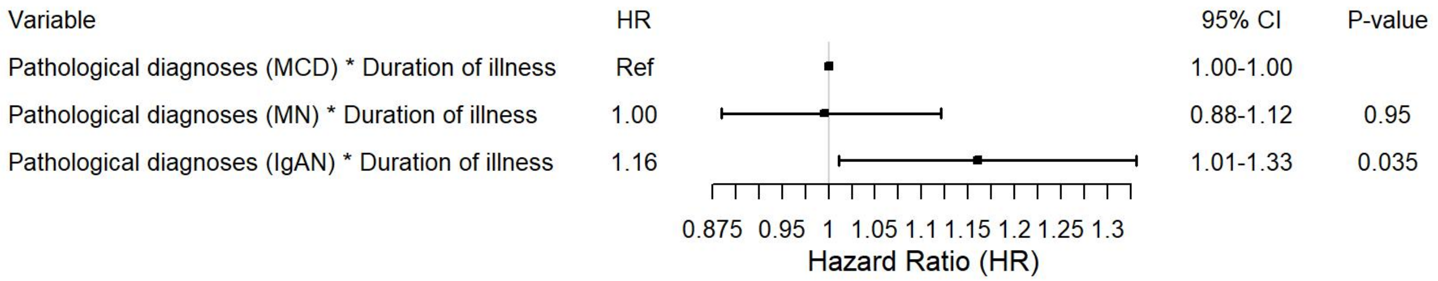

Figure S5 Forest Plot of the Multivariable Cox Regression Analysis on the Interaction Between Pathological Type and Disease Duration in Kidney Disease Progression

CI, confidence interval; HR, hazard ratio; IgAN, immunoglobulin A nephropathy; MN, membranous nephropathy; MCD, minimal change disease; Ref, reference

Table S1 Baseline Characteristics of the Included and Excluded Patients

Characteristics	Included Patients	Excluded Patients
Patients, n	703	482
Male sex	422 (60.0)	296 (61.4)
Age at biopsy, years	47.0 (38.0, 56.0)	44.0 (31.0, 54.0)
Hemoglobin, g/L	140.0 (125.0, 154.0)	135.0 (115.0, 152.6)
24-h urine total protein, g/d	3.8 (2.4, 5.1)	2.5 (1.4, 4.4)
Scr, μmol/L	73.1 (55.8, 93.8)	81.7 (64.0, 120.8)
eGFR[a], mL/min/1.73 m^2	100.1 (74.2, 119.5)	85.3 (38.2, 110.4)
Albumin, g/L	28.2 (22.2, 35.3)	31.6 (21.8, 42.6)
Pathological diagnoses, n		
MN	438 (62.3)	137 (28.4)
IgAN	110 (15.6)	81 (16.8)
MCD	60 (8.5)	43 (8.9)
FSGS	47 (6.7)	25 (5.2)
Other types of primary glomerular diseases[b]	48 (6.8)	15 (3.1)
Isolated Tubulointerstitial Lesions	0	29 (6.0)
Diabetic Nephropathy	0	109 (22.6)
Lupus Nephritis	0	15 (3.1)
Other types of secondary glomerular diseases[c]	0	28 (5.8)

eGFR, estimated glomerular filtration rate; FSGS, focal segmental glomerulosclerosis; IgAN, immunoglobulin A nephropathy; MN, membranous nephropathy; MCD, minimal change disease.

[a]GFR was calculated using the Chronic Kidney Disease Epidemiology Collaboration (CKD-EPI) equation.

[b]The other types of primary glomerular disease included endocapillary proliferative glomerulonephritis, non-IgA mesangial proliferative glomerulonephritis, pauci-immune crescentic glomerulonephritis, membranoproliferative glomerulonephritis, membranoproliferative glomerulonephritis, thin basement membrane nephropathy, Alport syndrome.

[c]The other types of secondary glomerular diseases included Henoch-Schönlein purpura nephritis, ANCA-associated vasculitis, amyloidosis-associated renal disease, IgG4-related kidney disease, among others.

Table S2 Therapy and outcomes of three major primary glomerulopathies

Therapy and Outcomes	MN (n=438)		IgAN (n=110)		MCD (n=60)	
	<3.5g/L (n=171)	≥3.5g/L (n=267)	<1g/L (n=25)	≥1g/L (n=85)	<3.5g/L (n=22)	≥3.5g/L (n=38)
Therapy after biopsy						
RASi alone	56 (32.7)	41 (15.4)	10 (40.0)	20 (23.5)	0	6 (15.8)
Steroid alone	16 (9.4)	27 (10.1)	6 (24.0)	33 (38.8)	17 (77.3)	28 (73.7)
IST alone	24 (14.0)	25 (9.4)	0	4 (4.7)	0	0
IST combined steroid	71 (41.5)	166 (62.2)	6 (24.0)	25 (29.4)	5 (22.7)	4 (10.5)
None	4 (2.3)	8 (3.0)	3 (12.0)	3 (3.5)	0	0
Remission of proteinuria[a]						
CR	84 (49.1)	132 (49.4)	20 (80.0)	43 (50.6)	22 (100.0)	32 (84.2)
PR	26 (15.2)	80 (30.0)	3 (12.0)	33 (38.8)	0	6 (15.8)
NR	53 (31.0)	37 (13.9)	2 (8.0)	9 (10.6)	0	0
Relapse	8 (4.7)	18 (6.7)	0	0	0	0
Kidney disease progression events[b]	22 (12.9)	39 (14.6)	1 (4.0)	13 (15.3)	1 (4.5)	3 (7.9)
ESKD	0	2 (0.7)	1 (4.0)	3 (3.5)	0	2 (5.3)
30% reduction of baseline eGFR	22 (12.9)	37 (13.9)	0	10 (11.8)	1 (4.5)	1 (2.6)

CR, complete remission; IgAN, immunoglobulin A nephropathy; IST, immunosuppressive therapy; MN, membranous glomerulonephritis; MCD, minimal change disease; NR, nonresponse; PR, partial remission; RASi, renin-angiotensin system inhibitors; Steroid, glucocorticosteroid.

[a]The definition of different outcomes: CR was characterized by a decrease in 24h-UTP excretion to ≤0.3 g/day, along with normalized serum albumin levels and stable renal function. PR was defined as ≥ 50% reduction in the 24h-UTP ratio and a final excretion of ≤3.5 g/day. NR was defined as 24h-UTP remaining above 3.5g/ day throughout follow-up, with a urinary protein reduction of less than 50%. Relapse was defined as new development of the 24h-UTP >3.5 g/d after CR or PR. Remission of proteinuria was referred to as CR and PR

[b]Kidney disease progression event was defined as a 30% decrease in eGFR during the follow-up period or the development of ESKD by at least 4 weeks. ESKD was defined as initiation of long-term dialysis, kidney transplant, or eGFR<15 mL/min/1.73 m^2. GFR was estimated according to the CKD Epidemiology Collaboration creatinine equation.

Table S3 The Interaction Between Pathological Type and Disease Duration in Proteinuria Outcome

**Interaction tests**	***p*-value**
Pathological diagnoses* 24-h UTP	0.81
Pathological diagnoses* LDL	0.77
Pathological diagnoses* IFTA	0.69
Pathological diagnoses* Arteriolar hyalinosis	0.33

IFTA, interstitial fibrosis and tubular atrophy; LDL, Low-density lipoprotein; UTP, urine total protein

Table S4 Univariable and multivariable analysis of variables for hazard of proteinuria outcome and kidney disease progression events in MN group

Variables	Proteinuria Outcome						Kidney Disease Progression					
	Univariable analysis			Multivariable analysis			Univariable analysis			Multivariable analysis		
	HR	95%CI	*p* value[a]	HR	95%CI	*p* value[a]	HR	95%CI	*p* value[a]	HR	95%CI	*p* value[a]
Age at biopsy, years	0.99	0.98-1.00	0.088				1.03	1.01-1.06	**0.012**	1.06	1.03-1.09	**<0.001**
Sex												
Male	Ref						Ref					
Female	0.94	0.75-1.17	0.56				0.61	0.36-1.04	0.07			
Family history of kidney disease	1.29	0.64-2.62	0.48				0.05	0.00-163.32	0.46			
Smoking history	1.28	1.01-1.63	**0.044**	1.18	0.92-1.51	0.19	3.79	2.25-6.38	**<0.001**	3.44	1.65-7.15	**0.001**
Alcohol history	1.27	0.99-1.62	0.06				2.82	1.69-4.70	**<0.001**	1.15	0.57-2.32	0.69
Duration of illness, months	1.01	0.99-1.04	0.24				1.06	1.03-1.08	**<0.001**	1.04	1.01-1.07	**0.013**
Comorbidities												
Hypertension	0.96	0.77-1.20	0.70				4.63	2.19-9.78	**<0.001**	3.22	1.45-7.16	**0.004**
Diabetes mellitus	1.29	0.97-1.72	0.08				1.11	0.54-2.25	0.78			
BMI, kg/m^2	1.00	0.96-1.03	0.82				1.05	0.97-1.13	0.20			
24-h urine total protein, g/d												
<3.5g/d	Ref						Ref					
≥3.5g/d	1.26	1.00-1.59	**0.049**	1.42	1.12-1.80	**0.004**	1.11	0.66-1.88	0.69			
eGFR[b], mL/min/1.73 m^2	1.00	1.00-1.00	0.25				1.00	0.99-1.01	0.47			
Hemoglobin, g/L	1.00	1.00-1.01	0.58				1.00	0.99-1.02	0.60			
Scr, µmol/L	1.00	1.00-1.00	0.08				1.00	1.00-1.01	**0.014**	1.00	1.00-1.01	0.08
Urea, mmol/L	1.00	0.99-1.02	0.59				0.99	0.93-1.06	0.86			
Uric acid, µmol/L	1.00	1.00-1.00	**0.005**	1.00	1.00-1.00	**0.004**	1.00	1.00-1.00	0.43			
Total cholesterol, mmol/L	0.98	0.93-1.03	0.37				1.09	0.97-1.23	0.16			
Triglycerides, mmol/L	0.93	0.88-0.98	**0.012**	0.93	0.88-0.99	**0.024**	1.00	0.91-1.10	0.985			
Low-density lipoprotein, mmol/L	0.94	0.89-0.99	**0.021**	0.94	0.89-0.99	**0.022**	1.00	0.94-1.07	0.995			
Albumin, g/L	1.01	0.99-1.03	0.36				0.94	0.90-0.98	**0.004**	0.96	0.92-1.00	**0.046**
IFTA[c]												
Absence	Ref						Ref					
Mild	0.47	0.36-0.61	**<0.001**	0.43	0.33-0.58	**<0.001**	0.43	0.22-0.84	0.014			

Moderate	0.33	0.14-0.75	**<0.001**	0.28	0.12-0.66	**0.004**	1.05	0.33-3.35	0.928			
Diffuse	0.36	0.09-1.48	0.16	0.38	0.09-1.56	0.18	0.00	$0.00\text{-}7.55\times10^{255}$	0.969			
Arteriolar hyalinosis	1.37	1.06-1.76	**0.015**	1.64	1.25-2.15	**<0.001**	1.68	0.75-2.99	0.075			
Crescent present	0.53	0.24-1.18	0.12				2.73	1.17-6.37	**0.020**	2.93	0.99-8.66	0.05
Nodular mesangial sclerosis present	0.77	0.55-1.07	0.12				2.84	1.66-4.88	**<0.001**	1.33	0.74-2.41	0.36
Glomerular obsolescence[c]												
Absence	Ref						Ref					
Mild	0.89	0.68-1.16	0.39				0.81	0.43-1.53	0.52			
Moderate	0.93	0.46-1.88	0.83				2.32	0.83-6.48	0.11			
Diffuse	0.63	0.16-2.53	0.51				0.00	$0.00\text{-}3.40\times10^{249}$	0.97			
Therapy after biopsy			**<0.001**			**<0.001**						
RAAS blockade alone	Ref			Ref			Ref					
Steroid alone	0.48	0.31-0.75	**0.001**	0.59	0.37-0.93	**0.023**	1.03	0.42-2.49	0.95			
IST alone/combined steroid	0.74	0.56-0.97	**0.032**	0.82	0.61-1.11	0.20	0.88	0.44-1.76	0.72			

Bold values indicate significant factors for the outcome event.

BMI, body mass index; CI, confidence interval; eGFR, estimated glomerular filtration rate; HR, hazard ratio; IFTA, interstitial fibrosis and tubular atrophy; MN, membranous glomerulonephritis; Ref, reference.

[a]Comparisons of *p* values in characteristics were performed using the Cox proportional hazard regression model. Parameters that were significant in the univariable analysis ($p < 0.05$) were included in this multivariate

[b]GFR was estimated according to the CKDEpidemiology Collaboration creatinine equation.

[c]IFTA and glomerular obsolescence were graded as absent (≤10% involvement), mild (10–30%), moderate (31–60%), or diffuse (>60%) based on the percentage of affected glomeruli or cortical tubulointerstitial area

Table S5 Univariable and multivariable analysis of variables for hazard of proteinuria outcome and kidney disease progression events in IgAN group

Variables	Proteinuria Outcome						Kidney Disease Progression					
	Univariable analysis			Multivariable analysis			Univariable analysis			Multivariable analysis		
	HR	95%CI	*p* value[a]	HR	95%CI	*p* value[a]	HR	95%CI	*p* value[a]	HR	95%CI	*p* value[a]
Age at biopsy, years	0.99	0.97-1.00	0.07				0.97	0.93-1.02	0.18			
Sex												
Male	Ref						Ref					
Female	0.88	0.59-1.32	0.54				1.26	0.44-3.60	0.67			
Smoking history	0.86	0.56-1.30	0.47				0.45	0.12-1.60	0.22			
Alcohol history	1.20	0.77-1.87	0.42				1.28	0.43-3.85	0.66			
Duration of illness, months	1.02	0.97-1.07	0.53				1.26	1.15-1.40	**<0.001**	1.00	0.78-1.31	0.96
Comorbidities												
Hypertension	1.23	0.82-1.83	0.32				1.41	0.49-4.04	0.522			
Diabetes mellitus	0.96	0.52-1.77	0.89				4.30	1.44-12.84	**0.009**	1.20	0.13-11.01	0.87
BMI, kg/m^2	1.01	0.96-1.05	0.84				1.03	0.91-1.15	0.68			
24-h urine total protein, g/d												
<1g/d	Ref						Ref					
≥1g/d	0.75	0.47-1.21	0.24				1.62	0.57-4.61	0.37			
eGFR[b], mL/min/1.73 m^2	1.00	1.00-1.00	0.75				0.98	0.97-1.00	**0.025**	1.01	1.00-1.03	0.18
Hemoglobin, g/L	1.00	0.99-1.01	0.80				0.96	0.93-0.99	**0.004**	1.00	0.96-1.04	0.81
Scr, μmol/L	1.00	1.00-1.00	0.59				1.01	1.00-1.01	**<0.001**	1.02	1.00-1.04	**0.039**
Urea, mmol/L	1.02	0.95-1.10	0.53				1.17	1.06-1.29	**0.001**	0.79	0.56-1.11	0.17
Uric acid, μmol/L	1.00	1.00-1.01	**0.004**	1.00	1.00-1.01	**0.004**	1.01	1.00-1.01	0.20			
Total cholesterol, mmol/L	0.98	0.84-1.14	0.76				0.54	0.31-0.95	**0.031**	0.86	0.34-2.17	0.74
Triglycerides, mmol/L	0.97	0.83-1.13	0.67				0.98	0.64-1.50	0.919			
Low-density lipoprotein, mmol/L	0.88	0.69-1.12	0.29				0.44	0.21-0.90	**0.024**	0.59	0.15-2.39	0.46
Albumin, g/L	1.01	0.98-1.04	0.68				1.00	0.93-1.08	0.998			

IFTA[c]						0.109
Absence	Ref			Ref		
Mild	0.76	0.39-1.49	0.43	0.34	0.07-1.75	0.196
Moderate	0.61	0.29-1.30	0.20	0.32	0.04-2.25	0.215
Diffuse	0.47	0.18-1.24	0.12	1.35	0.26-7.03	0.723
Arteriolar hyalinosis	1.04	0.67-1.61	0.85	1.38	0.47-4.00	0.558
Crescent present	0.84	0.57-1.26	0.40	2.08	0.65-6.65	0.215
Nodular mesangial sclerosis present	1.03	0.69-1.54	0.88	1.52	0.51-4.54	0.452
Glomerular obsolescence[c]						
Absence	Ref			Ref		
Mild	1.15	0.74-1.79	0.54	86592.84	$0.00\text{-}2.59\times10^{88}$	0.908
Moderate	1.36	0.77-2.40	0.29	227069.20	$0.00\text{-}6.80\times10^{88}$	0.900
Therapy after biopsy			0.115			
RAAS blockade alone	Ref			Ref		
Steroid alone	0.70	0.42-1.17	0.17	2.35	0.50-11.08	0.281
IST alone/combined steroid	0.60	0.35-1.02	0.06	0.58	0.08-4.12	0.587

Bold values indicate significant factors for the outcome event.

BMI, body mass index; CI, confidence interval; eGFR, estimated glomerular filtration rate; HR, hazard ratio; IFTA, interstitial fibrosis and tubular atrophy; IgAN, immunoglobulin A nephropathy; Ref, reference.

[a]Comparisons of *p* values in characteristics were performed using the Cox proportional hazard regression model. Parameters that were significant in the univariable analysis ($p < 0.05$) were included in this multivariate

[b]GFR was estimated according to the CKDEpidemiology Collaboration creatinine equation.

[c]IFTA and glomerular obsolescence were graded as absent (≤10% involvement), mild (10–30%), moderate (31–60%), or diffuse (>60%) based on the percentage of affected glomeruli or cortical tubulointerstitial area.

Table S6 The Interaction Between Pathological Type and Disease Duration in Kidney Disease Progression

**Interaction tests**	***p*-value**
Pathological diagnoses* Duration of illness	0.001
Pathological diagnoses* Hypertension	0.177
Pathological diagnoses* Nodular mesangial sclerosis present	0.355
